# Proceedings of the Tenth Annual Meeting of the American Medical Association

**Published:** 1857-07

**Authors:** 


### PROCEEDINGS OF THE TENTH ANNUAL MEETING OF THE AMERICAN MEDICAL ASSOCIATION.

(Condensed from the Nashville newspapers.)

NASHVILLE, May 5th, 1857.

THE Association met at 11 o'clock, A.M., in the Representative Hall of the State Capitol, the President, Dr. Zina Pitcher, of Michigan, in the chair, and upon his right Dr. W. K. Bowling, of Tennessee, one of the Vice-Presidents. Dr. Wm. Brodie, of Michigan, and Dr. R. C. Foster, of Tennessee, Secretaries, were present.

The meeting having been duly organized, Dr. C. K. Winston, chairman of the Committee of Arrangements, on behalf of the Committee, and of the Medical Profession of the city generally, extended a sincere and cordial welcome to the members of the Association in a few pertinent and appropriate remarks.

Upon the call of the roll the following delegates, permanent members, and members by invitation, numbering in all one hundred and forty-six, and representing nineteen States answered to their names.

*Connecticut.*—Charles Hooker.

*New Hampshire.*—Adoniram Smalley.

*New York.*—James R. Wood, D. M. Reese, George N. Burwell, and Alden March.

*Pennsylvania.*—Robley Dunglison, B. F. Schenck, Caspar Wister, and P. Cassidy.

*Georgia.*—Henry F. Campbell, C. R. Walton, N. F. Powers, A. H. Means, Joseph P. Logan, M. H. Oliver, Thomas S. Powell, J. Gordon Howard, R. D. Arnold, Geo. P. Padford, and Pike Brown.

*Alabama.*—G. M. Merriweather, W. P. Reese, A. F. Alexander, S. W. Clanton, W. H. Thornton, P. C. Winn, T. Stith Malone, W. J. Bass, G. D. Norris, J. F. Sowell, and J. W. Morris.

*Tennessee.*—Frank A. Ramsay, James Rodgers, R. O. Currey, B. B. Lenoir, J. L. C. Johnston, Jno. M. Boyd, Geo. R. Grant, T. A. Atchison, S. S. Mayfield, J. D. Kelley, T. L. Maddin, J. D. Winston, J. E. Manlove, G. A. L. Mayfield, Richard Owen, W. P. Jones, G. F. Smith, J. S. Burford, Jno. P. Ford, Robert C. Foster, John H. Callender, John H. Morton, A. H. Buchanan, James W. Hoyte, N. C. Perkins, John B. Lindsley, C. K. Winston, Paul F. Eve, permanent member; W. P. Moore, Milo Smith, Wallace Estill, B. W. Avent, H. H. Clayton, H. M. Whittaker, H. B. Manlove, T. M. Woodson, A. Ewing, Robert Martin, W. K. Bowling, P. S. Woodward, R. F. Evans, Thos. Lipscomb, M. Ransom, J. A. Long, Jno. M. Watson, W. D. Haggard, Jno. S. Park, D. B. Cliff, T. G. Kennedy, T. R. Jennings, Ira Conwell, W. H. Childress, W. A. Cheatham, J. F. Towns, J. M. Brannock, B. C. Jillson, P. W. Davis.

*Louisiana.*—S. O. Scruggs, Robert A. New, Cornelius Beard, and E. D. Fenner.

*Kentucky.*—Samuel Annan, R. W. Gaines, J. B. Flint, J. W. Singleton, R. J. Breckinridge, S. C. Porter, W. S. Chipley, S. M. Bemiss, L. G. Ray, W. A. Atchison, E. G. Davis, L. E. Almon, D. W. Yandell.

*Indiana.*—W. H. Byford, W. W. Hitt, Isaac Mendenhall, T. Bullard, N. Johnson.

*Illinois.*—J. C. H. Hobbs, A. H. Lace, James M. Steel, E. K. Carothers, T. K. Edmiston, W. A. Hills.

*Missouri.*—S. Pollak, E. S. Fraser, Jno. S. Moore, C. A. Pope.

*Michigan.*—A. B. Palmer, L. G. Robinson, Zina Pitcher, Wm. Brodie, L. H. Cobb, M. Gunn, Lewis Davenport, P. Cline, M. D. Stebbins.

*Iowa.*—Asa Horr, Wm. Watson, D. L. McGugin, J. C. Hughes.

*Ohio.*—Henry F. Koehne, J. M. Mosgrove, B. S. Browne, D. Ferris.

*Wisconsin.*—Hays McKinley, J. K. Bartlett.

*South Carolina.*—E. R. Henderson, M. S. Moore, R. W. Gibbs, R. S. Bailey.

*Mississippi.*—F. B. Shuford, J. S. Cain.

*Arkansas.*—F. Grundy M'Gavock.

On motion of Dr. C. K. Winston, Drs. Felix Robertson, J. Shelby, and J. Overton, were made permanent members of the Association.

The usual recess to allow each State Delegation to select a member to serve on the Committee on Nominations having been taken, the following list was reported:

Charles Hooker, Conn.; Adoniram Smalley, N. H.: W. W. Hitt, Ind.; J. R. Bartlett, Wis.; James R. Wood, N. Y.; A. B. Palmer, Mich.; J. S. Moore, Mo.; J. K. Edmiston, Ill.; R. J. Breckinridge, Ky.; F. Grundy M'Gavock, Ark.; B. S. Brown, Ohio; R. W. Gibbs, S. C.; W. P. Reese, Ala.; F. B. Shuford, Miss.; Richard M. Cooper, N. J.; S. O. Scruggs, La.; P. Cassidy, Penn.; Thomas S. Powell, Ga.; J. B. Lindsley, Tenn.; Asa Horr, Iowa.

On motion of Dr. Charles Hooker, it was resolved that the President, Dr. Z. Pitcher, be now requested to deliver his annual address.

The President then delivered his address, which, after the customary thanks ot the Association, was referred to the Committee on Publication.

Judge Catron, of the United States Supreme Court, being present, was invited to a seat on the stand.

The Report of the Committee on Publication was next read by Dr. Caspar Wister, of Pennsylvania, and also that of the Treasurer, Dr. Wister, which, after being adopted, were referred to the Committee on Publication.

The President informed the Association that Dr. F. Campbell Stewart, of New York, Dr. Alden March, of New York, Dr. Isador Glück, of New York, and Dr. J. Pancoast, of Pennsylvania, had been appointed to represent the Association in foreign scientific bodies.

The Committee on Prize Essays requested further time.

The only report received from the several committees on Medical Topography and Epidemics, was one from Dr. Peregrine Wroth, of Maryland, which, with accompanying reports from Drs. A. M. White and Edmund E. Waters, was referred to the Committee on Publication.

Dr. Arnold presented a report from Dr. J. F. Posey, of Georgia, but subsequently withdrew it, for the purpose of making an abstract of it.

The Committee on Nominations now appeared, and reported the following officers of the Association for the ensuing year:

*President*—Dr. Paul F. Eve, of Tennessee.

*Vice-Presidents*—Drs. R. J. Breckinridge, of Kentucky; D. M. Reese, of New York; W. H. Byford, of Indiana; and Henry F. Campbell, of Georgia.

The nominees were duly elected, and Drs. Wister, Arnold, and McGugin, were appointed a Committee to conduct the newly-elected officers to their respective chairs. But the President elect being absent, the Association adjourned.

SECOND DAY.

The Association having met pursuant to adjournment, and the minutes read and adopted, the newly-elected officers were inducted to their respective seats.

Dr. Eve, in taking the presidential chair, made a brief and appropriate address.

Dr. Winston read the names of additional delegates.

The President appointed Drs. Currey, Grant, and Evans, a Committee on Voluntary Contributions.

Dr. Arnold read an abstract from Dr. Posey's Report, which was referred.

Numerous States were called upon for reports upon Medical Topography and Epidemics; but there was either no response, or the committees asked further time.

A communication was received from the Southern Methodist Publishing House, inviting the members of the Association to visit that establishment, and the invitation accepted.

Dr. Lindsley read a communication from the Medical Association of Washington City, inviting the Association to hold their next Annual Meeting in that city. Referred to the Committee on Nominations.

A resolution of thanks to the late President, for the able manner in which he presided over the meetings of the Association, was unanimously adopted.

The reports of Special Committees for 1856–7 being next in order, were called for, and the following presented:

[We omit the enumeration of committees from which no reports were received.]

*Causes of Infant Mortality, &c.* Dr. D. Meredith Reese, of New York, read an abstract of his report, which was referred to the Publishing Committee.

*Blending and Conversion of the Types of Fever.* Dr. C. G. Pease, of Wisconsin. Report and communication sent, but not received. (Subsequently received, and referred to the Committee on Voluntary Contributions.)

*Use of Cinchona in Malarious Diseases.* Dr. F. Hinkle, of Pennsylvania. Report referred to Publishing Committee.

*Nervous System in Febrile Diseases.* Dr. H. F. Campbell, of Georgia. Report; verbal abstract given; referred.

*Medical Topography and Fauna of Washington Territory.* Dr. G. Suckley, United States Army. Report presented and referred.

*Flora of Washington and Oregon Territories.* Dr. Jas. Cooper, of New Jersey. Report presented and referred.

*Etiology and Pathology of Epidemic Cholera.* Dr. T. W. Gordon, of Ohio. Partial report presented and referred.

[Between the calling of some of the reports, other subjects were introduced and discussed, but none of any special interest, except the following:]

The Secretary read a protest, signed by Drs. Richard Arnold, J. G. Howard, P. Brown, and G. P. Padford, against admitting the delegates from the Oglethorpe Medical College. A warm discussion ensued, and the whole matter was finally referred to a committee of three, to be appointed by the chair.

The chair appointed Drs. Wister, of Penn., Bemiss, of Ky., and Gibbs, of S. C., who subsequently reported in favor of admitting the delegates mentioned. The report was adopted.

Resolutions of regard to the memories of Drs. Grafton, of Miss., Robert M. Porter, of Tenn., and J. L. Mothersett, of Indiana, were presented respectively by Dr. Dunglison, Dr. Bullard, and the Secretary, all of which were unanimously adopted.

A communication was received from the Connecticut Medical Society, asking that the time for holding the meetings of the Association in Northern cities be changed to a later period in the year. Referred to the Committee on Nominations, with instructions to report.

THIRD DAY.

The Association having met, the minutes read and adopted, Dr. Hoyt, from the Committee on Arrangements, read the names of additional delegates.

Dr. Currey, from the Committee on Voluntary Contributions, reported in favor of publishing the following papers in the Transactions of the Association:

1. *A new Principle of Diagnosis in Dislocations of the Shoulder-Joint.* By Dr. L. A. Dugas, of Georgia.

2. *Medical Statistics of Washington Territory.* By Dr. George Suckley, U. S. Army.

3. *Medical Flora of Washington and Oregon Territories.* By Dr. G. A. Cooper.

Dr. Lindsley, from the Nominating Committee, submitted the following report:

Committee on Nominations beg leave to report:

*Secretaries.*—Robert C. Foster, of Tenn.; A. J. Semmes, of Washington City.

*Treasurer.*—Caspar Wister, of Philadelphia.

For the next place of meeting, Washington City.

STANDING COMMITTEES.

*Committee of Publication.*—Francis G. Smith, of Philadelphia, Chairman; Caspar Wister, of Philadelphia; R. C. Foster, of Nashville; A. J. Semmes, of Washington City; Samuel L. Hollingsworth, of Philadelphia; Samuel Lewis, of Philad.; H. F. Askew, of Delaware.

*Committee on Prize Essays.*—Grafton Tyler, of Georgetown, D. C., Chairman; J. C. Hall, of D. C.; J. F. May, of D. C.; Thomas Miller, of D. C.; A. J. Semmes, of D. C.; Joshua Riley, of D. C.; W. J. C. Duhamel.

*Committee of Arrangements.*—Harvey Lindsley, Chairman; W. J. C. Duhamel, Cornelius Boyle, P. H. Coolidge, G. M. Dove, A. Y. P. Garnett, Wm. P. Johnston, of D. C.

*Committee on Medical Education.*—G. W. Norris, of Philadelphia, Chairman;

A. H. Luce, of Ill.; E. R. Henderson, of S. C.; G. R. Grant, of Tenn.; T. S. Powell, of Ga.

*Committee on Medical Literature.*—A. B. Palmer, of Detroit, Chairman, A. F. Alexander, of Ala.; J. M. Mosgrove, of Ohio; P. Cassidy, of Penn.; S. Pollak, of Missouri.

*Vacancies in Committee on Medical Topography and Epidemics.*—T. B. Shuford to fill the vacancy caused by the death of Dr. Grafton, of Miss. C. W. Parsons to fill the vacancy caused by resignation of Joseph Mauran, of Rhode Island.

SPECIAL COMMITTEES.

*Spontaneous Umbilical Hemorrhage of the Newly Born.*—J. Foster Jenkins, of New York.

*Influence of Marriages of Consanguinity upon Offspring.*—Dr. Bemiss, of Ky.

*Functions of different portions of the Cerebellum.*—E. Andrews, of Ill.

*Causes of the Impulse of the Heart and the agencies which influence it in health and disease.*—J. W. Corson, of New York City.

*Treatment of the results of Obstructed Labor.*—J. Marion Sims, N. Y.

*Treatment best adapted to each variety of cataract, with the method of operation, place of election, time, age, &c.*—Mark Stephenson, of N. Y.

*Human, Animal, and Vegetable Parasites.*—Joseph Leidy, of Phila.

*Best substitutes for Cinchona and its preparations in the treatment of Intermittent Fever, &c.*—B. S. Woodward, of Ind.

*Intimate structure and pathology of the Kidney.*—Charles E. Isaacs, of N. Y.

*Etiology and Pathology of Epidemic Cholera.*—T. W. Gordon, Georgetown, Broome Co., Ohio.

*Inflammation of Cervix Uteri.*—Henry H. Miller, of Louisville, Ky.

*On Milk Sickness.*—W. H. Byford.

*Best means of causing an increase of the number of Essays.*—Drs. Leidy, Woods, and Meigs, of Pa.

*Changes produced in composition and properties of Milk.*—N. S. Davis, of Ill.

*Stomatitis Materna.*—D. C. McGugin, Iowa.

*On Criminal Abortion, with a view to its general suppression.*—H. N. Storer, of Boston.

The Committee recommend the amendment of the third article of the Constitution, in relation to *meetings*, by inserting after the words, "first Tuesday in May," the words, "*or the first Tuesday in June*," and also by inserting after the words, "shall be determined," the words, "*with the time of meeting.*"

*Special Committee on the present state of science, as regards the pathology and therapeutics of the reproductive organs of the female.*—D. Fordyce Barker, of New York.

*On Moral Insanity.*—D. Meredith Reese, M.D., New York.

*On Calculi and Diseases of the Urinary Organs, in Iowa, Minnesota, and Nebraska.*—Dr. J. C. Hughes, of Keokuk.

*On the nature, tendency, and general treatment of Syphilitic Buboes.*—Moses Gunn, of Detroit.

*On Medical Education*—(By Dr. Currey's resolution, reported below).—James R. Wood, of New York; George R. Grant, Memphis, Tenn.; John Watson, New York; C. B. Nottingham, Macon, Ga.; R. La Roche, Philadelphia, Penn.

*To fill a vacancy in the Committee on Medical Topography and Epidemics.*—Dr. J. L. Cabell, Charlottesville, Va.

The report of the Committee was adopted.

Dr. Pitcher offered the following resolution, which was unanimously adopted:

*Resolved,* That a Committee of three be appointed, of which the President of the Association shall be Chairman, to communicate with the Surgeon-General of the Army, the chief of the Medical Bureau of the Navy, and the Secretary of the Treasury of the United States, with a view to secure the concurrence of these departments of the Federal Government, so that its contributions to the Medical Topography, the Vital Statistics, and the Sanitary Police of the nation, may be made tributary to the labors of this Association.

The Chairman appointed as such committee, Drs. Z. Pitcher, of Michigan, and R. H. Coolidge, of Kansas.

A Resolution was introduced by Dr. Yandell in reference to the admission of delegates from medical schools, having less than six professors, and holding more than one session per year.

After some discussion Dr. Currey offered the following as a substitute, which was finally adopted.

*Whereas,* The subject of Medical Education has been committed at each annual Session to standing committees, and various suggestions have been proposed which the Association has adopted, and recommended to private instructors and to the Medical Colleges.

*Resolved,* That a committee of five be appointed by the Committee on Nominations, as a Special Committee, to be composed of members who are in no respect connected with any Medical School, to devise *a System of Medical Instruction,* to be presented for the consideration of this Association at its Annual Session in 1858.

*Resolved,* That the proposed system shall set forth a uniform basis upon which our Medical Institutions shall be organized, as well as have reference to the best mode of securing the Preparatory Medical Instruction to the Student; and that consequently the legitimate subjects to be embraced in said system will include Primary Medical Schools; the number of Professorships in Medical Colleges, the length and number of terms during the year, the requisite qualifications for graduation, and such other subjects of a general character as to give uniformity to our Medical System, and preserve harmony and friendly intercourse in the ranks of the profession.

*Resolved,* That, upon the adoption of the proposed system by the Association, all Institutions which may conform to it shall be entitled to representation at the Annual Sessions of this Association, and none others.

Dr. Bowling, chairman of the Committee on Prize Essays, submitted the report of said committee, as follows:—

The Committee on Prize Essays report that four essays have been received, each possessing great merit.

The committee selected the two following essays for the two prizes provided for at the last meeting of this Association:

1. One entitled "The Excreto-Secretory System of Nerves: its Relations to Physiology and Pathology." Motto: "*Observation becomes experiment when used in severe processes of induction.*" Signed, Henry Frazer Campbell, Georgia.

2. "Experimental Researches relative to the Nutritive Value and Physiological Effects of Albumen, Starch, and Gum, when singly and exclusively used as Food." Motto:

> "Quum sequimus? quone in jubes? ubi ponere sedis?
> Da pater augurium, atque animis illabere nostris!"

Signed, William A. Hammond, M.D., Assistant Surgeon, U. S. Army.

The Committee on Voluntary Contributions reported in favor of the publication in the Transactions of the Association of the following paper: "On the Blending and Conversion of Types in Fever." By C. S. Pease, M.D., of Wisconsin. The report was adopted.

The amendments to the Constitution proposed by Dr. Stocker, of Pa., at the last annual session, were taken up and laid on the table.

Dr. Lindsley offered the following amendment to the Constitution, which was seconded by Dr. Gunn:

In Art. II. omit the words "medical colleges," and also the words "the faculty of every regularly constituted medical college or chartered school of medicine shall have the privilege of sending two delegates."

The amendment lies over until the next meeting of the Association, under a rule of the organization.

On motion of Dr. Palmer, the resolutions reported at the last annual meeting of the Association, by the Committee on Plans of Organization for State and County Medical Societies, were taken up and adopted.

After the transaction of some other unimportant matters the Association adjourned, *sine die.*

